# Psychometric Validation of the Spanish Version of the Luxembourg Workplace Mobbing Scale (LWMS): Structural Equation Modeling, and Item Response Theory Evidence

**DOI:** 10.3390/bs16040615

**Published:** 2026-04-21

**Authors:** Jonatan Baños-Chaparro, Andrei Franco-Jimenez, Javier Hildebrando Espinoza Escobar, Tomás Caycho-Rodríguez, Fabio Cesar Saldivar Celis

**Affiliations:** 1 Programa Académico de Psicología, Facultad de Ciencias de la Salud, Universidad Privada Norbert Wiener, Lima 15046, Peru; 2 Facultad de Psicología, Universidad Nacional San Luis Gonzaga, Ica 11000, Peru; andrei.franco@unica.edu.pe; 3 Programa Académico de Derecho, Facultad de Derecho, Universidad Privada Norbert Wiener, Lima 15046, Peru; javier.espinoza@uwiener.edu.pe (J.H.E.E.); a2023202209@uwiener.edu.pe (F.C.S.C.); 4 Facultad de Psicología, Universidad Científica del Sur, Lima 15046, Peru; tcaycho@cientifica.edu.pe

**Keywords:** workplace mobbing, mental health, adult, reliability, item response theory

## Abstract

**Introduction**: Workplace mobbing is a psychosocial risk factor associated with adverse mental health outcomes, including depression, anxiety, and suicidal ideation. Accurate assessment of this phenomenon is essential for both research and applied settings; however, validated brief instruments in Spanish remain limited. The Luxembourg Workplace Mobbing Scale (LWMS) is a short measure with sound psychometric properties that allows efficient evaluation of exposure to workplace mobbing. **Objective**: Translation and validation of the LWMS into Spanish in adults. **Methods**: A total of 345 adults (51.3% women) participated, completing a sociodemographic questionnaire and psychological instruments. Statistical analyses were conducted using structural equation modelling and item response theory. **Results**: The LWMS demonstrated adequate content validity; a unidimensional structure (CFI = 0.99, RMSEA = 0.04 [90% CI: 0.001, 0.092], SRMR = 0.02); and reliability (ω = 0.79, H = 0.86 and r_xx_ = 0.78). In addition, significant associations were found with depressive symptoms (r = 0.37, *p* = 0.001), generalised anxiety (r = 0.38, *p* = 0.001), and suicidal ideation (r = 0.27, *p* = 0.001). Item 2 showed the highest discrimination and information, and the scale proved to be accurate at higher levels of workplace mobbing. **Conclusions**: The Spanish version of the LWMS shows solid evidence of validity and reliability, supporting its use as a brief and precise instrument for assessing workplace mobbing in adult populations. Its strong psychometric performance and clinical relevance make it suitable for research, screening, and preventive interventions in occupational settings.

## 1. Introduction

Workplace mobbing is a form of workplace harassment characterised by hostile, repeated and prolonged behaviours that place the affected individual in a position of defencelessness and power asymmetry (Leymann, 1996; Meng, 2025). Unlike occasional interpersonal conflicts, mobbing involves a systematic and sustained dynamic over time, whose purpose—explicit or implicit—is to marginalise, discredit or force the victim out of the work environment (Meng, 2025). These behaviours include threats, humiliation, intimidation, ridicule, social isolation and the assignment of degrading tasks (Grotto-de-Souza et al., 2023). It differs from ordinary workplace conflicts due to its systematic and repetitive nature (Tatar & Yüksel, 2019), making it a particularly harmful form of psychological violence at work because of its cumulative potential and its progressive impact on professional identity and employees’ emotional well-being. Although the boundaries between workplace mobbing, workplace bullying, and related forms of workplace harassment are not fully consistent and are sometimes used interchangeably, the term mobbing is more commonly used in European research traditions (Chirilă & Constantin, 2013; Crawshaw, 2009).

Globally, it is estimated that more than one in five workers has experienced some form of violence or harassment at work (International Labour Organization, 2022). Within this spectrum, mobbing represents a specific form of organisational psychological violence, with prevalence rates exceeding 20% in certain contexts, particularly in sectors such as healthcare, education and social services (Ciby & Raya, 2015). These figures not only demonstrate its high frequency but also underscore its relevance as a structural public health concern and a significant psychosocial occupational risk.

Beyond its prevalence, exposure to workplace mobbing is associated with significant deterioration in mental health, due to its persistent nature, the power asymmetry underpinning it and the chronic stress it generates (Leymann, 1996; Rincon-Hoyos et al., 2025). The repetitive and prolonged nature of these behaviours fosters cumulative psychological wear, negatively affecting self-esteem, perceived self-efficacy and sense of workplace belonging (Meng, 2025; Rincon-Hoyos et al., 2025). Consistently, higher exposure to mobbing has been linked to elevated levels of depressive symptoms and generalised anxiety (Machado et al., 2021). Moreover, exposed workers may develop symptomatology consistent with post-traumatic stress disorder, particularly when experiences involve direct threats, systematic isolation or prolonged reputational harm (Tatar & Yüksel, 2019). Accordingly, several studies have documented an increased risk of suicidal behaviours among individuals exposed to mobbing, including suicidal ideation and suicide attempts (Ayala-Burboa et al., 2024; Pompili et al., 2008). These findings suggest that mobbing should not be conceptualised solely as an organisational or workplace climate issue, but rather as a psychosocial determinant with clinically relevant consequences, highlighting its relevance for mental health research.

From a behavioral science perspective, workplace mobbing can be conceptualised as a chronic interpersonal stressor embedded within social and organisational contexts (Meng, 2025). According to stress and coping theory, repeated exposure to aversive social interactions may activate maladaptive cognitive and emotional responses, reinforcing negative appraisal patterns and emotional dysregulation (Rincon-Hoyos et al., 2025; Machado et al., 2021). Additionally, cognitive vulnerability models suggest that persistent exposure to humiliation and exclusion may contribute to the development of dysfunctional beliefs, such as perceived helplessness and loss of control, which are key mechanisms underlying anxiety, depression, and suicidal ideation (Pompili et al., 2008; Ayala-Burboa et al., 2024). Thus, mobbing can be understood not only as an organisational phenomenon but also as a behavioral process with significant implications for mental health (Grotto-de-Souza et al., 2023).

In Latin America, where informal employment affects approximately half of the workforce (International Labour Organization, 2024), the study of mobbing acquires particular importance. In Peru, around 70% of workers are employed under informal conditions (Astorima Huanca, 2024; Instituto Peruano de Economía, 2025), which may hinder reporting, institutional regulation and the early identification of harassment. Job precariousness, the absence of formal protective mechanisms and rigid hierarchical dynamics may increase vulnerability to persistent harassment behaviours. Such contextual factors may not alter the core definition of mobbing but may shape how it is perceived and reported in practice across cultural and socio-economic contexts (Salin et al., 2019). In this context, Spanish-language instruments are needed to assess exposure to mobbing in a valid, reliable and efficient manner, both in epidemiological research and in clinical and organisational settings.

However, the assessment of mobbing in the region faces important methodological challenges. Available adaptations differ across countries, limiting cross-cultural comparability and the accumulation of systematic evidence (López & González-Trijueque, 2022). Furthermore, widely used scales such as the Negative Acts Questionnaire-Revised (NAQ-R) and the Leymann Inventory of Psychological Terrorisation (LIPT) may be lengthy for population-based studies or brief organisational assessments (Córdova Campaña et al., 2022; Steffgen et al., 2019). The length of these instruments may increase respondent fatigue and reduce their applicability in large-scale research or clinical screening contexts. These limitations reinforce the need for brief instruments with robust psychometric properties.

In response to this need, the Luxembourg Workplace Mobbing Scale (LWMS) represents a promising alternative for assessing workplace mobbing. It is a brief five-item instrument assessing core behavioural manifestations associated with workplace mobbing, such as being unfairly criticised, ignored or excluded, assigned absurd or degrading tasks, ridiculed in front of others and experiencing persistent conflicts with colleagues, which, when occurring over time, may reflect underlying mobbing dynamics as part of a broader pattern of negative interactions (Steffgen et al., 2019). The LWMS has demonstrated a unidimensional structure, suggesting that different forms of mobbing are closely related given their high empirical overlap (Marsh et al., 2011). In addition, evidence of construct validity has been reported, with positive associations with psychological stress and burnout, and negative associations with job satisfaction and workplace respect. It has also shown appropriate functioning in German, French and Luxembourgish versions, supporting its utility in comparative and multicultural research (Steffgen et al., 2019). However, to date, no published psychometric validation of a Spanish version is available, limiting its use in Spanish-speaking contexts.

For these reasons, the present study aimed to evaluate the psychometric properties of the Spanish version of the LWMS in a sample of Peruvian workers, within a specific cultural context, addressing the lack of validated brief instruments for assessing workplace mobbing in Spanish-speaking contexts. Specifically, the study sought to examine (a) its factorial structure using confirmatory factor analysis, (b) its internal consistency using Bayesian reliability approaches, (c) item functioning through item response theory, and (d) its convergent validity based on associations with depressive symptoms, generalised anxiety, and suicidal ideation. It was hypothesised that the LWMS would show a unidimensional structure, adequate reliability, and significant associations with these clinically relevant indicators. By integrating evidence derived from covariance-based models and probabilistic measurement models, the study adopts a multimethod psychometric approach that enhances the precision and interpretability of the scale scores.

## 2. Materials and Methods

### 2.1. Participants

The research followed an associative, comparative, quantitative, and cross-sectional design. The inclusion criteria were: (a) being of Peruvian nationality, (b) being between 18 and 60 years old, and (c) currently working in the public or private sector. Individuals who did not meet these criteria were excluded from the study.

In total, 345 Peruvian adults from the general population participated. The sample was balanced by sex, with 51.3% women and 48.7% men. The mean age was 33 years (SD = 9.4), ranging from 18 to 60 years. Regarding sociodemographic characteristics, most participants identified as single (72.5%), followed by married (24.3%), divorced (2.6%), and widowed (0.6%). In terms of educational level, most had completed university studies (40.3%), followed by postgraduate education (26.4%) and incomplete university studies (12.7%). With respect to employment conditions, the most common type of contract was indefinite-term (54.2%), followed by fixed-term (45.8%). Most participants worked full-time (81.7%), while smaller proportions reported part-time work over 4 h (9.8%) or up to 4 h (8.5%). In terms of job tenure, 28.9% reported 5 years or more, 26.1% between 1 and 2 years, and 24.1% less than 6 months. Finally, regarding changes in the number of employees at the workplace, 35.9% reported no change, while others indicated a slight increase (32.7%), a significant increase (14.5%), a slight decrease (11.6%), or a significant decrease (5.3%).

### 2.2. Measures

#### 2.2.1. Demographic Information

A brief questionnaire was administered to collect participants’ information, including sex, age, marital status, educational level, employment sector, type of contract, work schedule, job tenure, and changes in the number of employees at the workplace.

#### 2.2.2. Luxembourg Workplace Mobbing Scale (LWMS)

The LWMS is a brief instrument designed to assess workplace mobbing (Steffgen et al., 2019). It consists of five items, with response options ranging from never (1), almost never (2), sometimes (3), often (4), to almost always (5). The total score ranges from 5 to 25, with higher scores indicating greater exposure to workplace mobbing.

For the Spanish adaptation of the instrument, the back-translation procedure was employed. In the first phase, a professional bilingual translator with experience in psychological and organisational terminology independently translated the scale from English into Spanish. Subsequently, a second translator carried out the back-translation from Spanish into English. Following this process, a joint review was conducted by both translators and the research team to reach consensus and validate the final version. During this stage, discrepancies between the original and back-translated versions were systematically analysed, focusing on semantic equivalence, conceptual consistency, and cultural appropriateness. When discrepancies emerged, they were resolved through discussion and consensus, prioritising conceptual over literal equivalence. Next, five expert judges in organisational and clinical psychology (with demonstrated experience in psychometrics and workplace mental health) evaluated the items according to three essential criteria: relevance, representativeness, and clarity. In addition, judges were asked to assess the cultural adequacy and contextual appropriateness of each item for the Peruvian occupational setting. Finally, prior to formal administration, a pilot test was conducted with ten adults to assess the comprehension and clarity of the items in the target population. Participants were selected to represent varying educational backgrounds, and brief cognitive probing was conducted to verify item comprehension and interpretability. No comments requiring modification were received, suggesting adequate linguistic clarity and acceptability of the items in the target context. Table 1 presents both the original English version and the Spanish translation.

#### 2.2.3. Frequency of Suicidal Ideation Inventory (FSII)

It is an inventory that measures the frequency of suicidal ideation over the past year based on five items. Responses are provided on a Likert-type scale ranging from 1 (never) to 5 (almost every day). The sum of the items yields a total score ranging from 5 to 25 points. Higher scores indicate greater frequency of suicidal ideation. The Peruvian adaptation was used in this study, which showed acceptable reliability (ω = 0.94) (Baños-Chaparro et al., 2021).

#### 2.2.4. Patient Health Questionnaire-2 (PHQ-2)

The PHQ-2 is a brief two-item questionnaire that assesses depressive symptoms over the past two weeks. Each item is rated on a four-point scale from 0 (not at all) to 3 (nearly every day). The total score ranges from 0 to 6, with higher scores indicating greater depressive symptomatology. The Peruvian adaptation was used, and this study reported good reliability (ω = 0.73) (Villarreal-Zegarra et al., 2023).

#### 2.2.5. Generalized Anxiety Disorder-2 (GAD-2)

The GAD-2 is a short scale assessing generalized anxiety over the past two weeks through two items. Each item is rated on a four-point scale from 0 (not at all) to 3 (nearly every day). The total score ranges from 0 to 6, with higher scores reflecting greater levels of generalized anxiety. The Peruvian adaptation was used, and this study reported adequate reliability (ω = 0.88) (Franco-Jimenez & Nuñez-Magallanes, 2022).

### 2.3. Procedure

Data collection was conducted online through the administration of a web-based survey between May and July 2025. The questionnaire was hosted on Google Forms and disseminated via the researchers’ social media platforms, which facilitated access to a broad range of potential participants. The survey included detailed information regarding the aims of the study, the anonymous nature of participation, the exclusive academic use of the data collected, the procedures for data processing, and informed consent, which participants were required to provide before proceeding with the questionnaire.

Internet-based surveys offer several methodological advantages, including greater accessibility to the study sample, systematic control of responses, the possibility of using multiple dissemination channels, and an efficient and cost-effective administration process, optimising both time and resources during data collection (Gosling & Mason, 2015).

### 2.4. Statistical Analysis

The statistical analysis was conducted in a sequential and systematic manner using RStudio software (version 4.3.2), following a multi-stage approach aimed at the comprehensive psychometric evaluation of the instrument.

In the first stage, a thorough descriptive analysis of the items was performed to examine their statistical behaviour and preliminary quality. Measures of central tendency and dispersion (mean and standard deviation) were calculated, along with distributional indicators such as skewness and kurtosis. In addition, a polychoric correlation matrix was estimated, taking into account the ordinal nature of the items, together with corrected item–total correlations, adopting values greater than 0.30 as the criterion for adequacy (Kline, 2023). Complementarily, content validity was assessed using Aiken’s V coefficient, with values above 0.70 considered acceptable, in accordance with previous methodological recommendations (Roebianto et al., 2023). Additionally, to evaluate the potential presence of common method bias, Harman’s single-factor test was conducted (Kline, 2023). A single-factor solution did not account for the majority of the total variance, suggesting that common method variance was not a substantial concern in this study.

In the second stage, a confirmatory factor analysis (CFA) was conducted to examine the internal structure of the instrument. Given the ordinal nature of the data, the Weighted Least Squares Mean and Variance adjusted (WLSMV) estimator was employed, as it is considered appropriate for this type of measurement. Model fit was evaluated using multiple goodness-of-fit indices, including the Comparative Fit Index (CFI), the Root Mean Square Error of Approximation (RMSEA), and the Standardised Root Mean Square Residual (SRMR). Values of CFI greater than 0.95 and RMSEA and SRMR values below 0.08 were used as criteria for acceptable model fit (Jordan Muiños, 2021). Additionally, the magnitude of the standardised factor loadings was examined, with values exceeding 0.30 considered adequate (Kline, 2023).

The third stage focused on estimating the reliability of the instrument from a contemporary perspective. The Bayesian omega coefficient (ω), H coefficient, and empirical reliability (r_xx_). This approach allowed for more precise and robust estimates of score consistency, overcoming the limitations of traditional reliability coefficients, as recommended in the specialised literature (Baños-Chaparro & Caycho-Rodríguez, 2024; Gunnell, 2020; Pfadt et al., 2022).

In the fourth stage, a covariance-based structural equation model (CB-SEM) was estimated to examine the relationships among the latent variables in the study. The robust maximum likelihood (MLR) estimator was employed, and overall model fit was assessed using the CFI, RMSEA, and SRMR indices, following the same acceptance criteria previously established (Jordan Muiños, 2021). The magnitude of the associations was interpreted according to the cut-off values proposed by Cohen (1988), considering small = 0.10, moderate = 0.30, and large = 0.50 effects.

Finally, in the fifth stage, a two-parameter Item Response Theory (IRT) model (2PL) was estimated. This model considered the discrimination parameter (*a*), which indicates the ability of an item to distinguish between individuals with low and high levels of the latent trait (θ), with values greater than 1 regarded as acceptable (Baker & Kim, 2017). The second parameter was the difficulty parameter (*β*), which represents the probability of responding to adjacent response categories across the continuum of the latent trait (θ). The model was specified using the Graded Response Model (GRM), and key assumptions were examined, including unidimensionality, local independence assessed through the standardised LD-X^2^ statistic (LD-X^2^ < 10), and monotonicity, evaluated using a non-parametric IRT Mokken model with the calculation of the critical statistic (*crit* < 0.40) (Sijtsma & van der Ark, 2017; Stover et al., 2019).

Information estimation was conducted using the Item Information Curves (IIC) and the Test Information Curve (TIC). To ensure that the model was correctly specified, global model fit was evaluated using the C^2^ statistic, which is recommended for ordinal IRT models, along with fit indices such as CFI > 0.90 (Cai et al., 2023; Lubbe & Schuster, 2019; Monroe & Cai, 2015). In addition, RMSEA_2_ and SRMR were considered indicators of adequate fit (RMSEA_2_ = 0.089, SRMR = 0.05) and excellent fit (RMSEA_2_ = 0.05, SRMR = 0.027). Given that the number of response categories may influence ordinal IRT models, the adjusted RMSEA_2_/(K − 1) and SRMR/(K − 1) indices were applied in accordance with the recommendations of Maydeu-Olivares and Joe (2014). To further support these findings, local item fit was assessed for each item using the S-X^2^ index and RMSEA.S-_X_^2^ < 0.06 (Bean & Bowen, 2021; Kang & Chen, 2008), as well as the infit and outfit statistics, with values between 0.50 and 1.5 indicating good fit (Wright & Linacre, 1994).

### 2.5. Ethical Considerations

The research was conducted in strict compliance with international and national ethical standards governing psychological and social science research. Prior to data collection, all participants were informed about the nature and purpose of the study and provided their informed consent electronically. Participation was entirely voluntary, the survey was administered in an anonymous format, and robust measures were implemented to ensure the confidentiality and secure handling of all collected data, in line with established ethical guidelines (Hilbig et al., 2022). Furthermore, the study protocol underwent formal ethical review and received approval from the Ethics Committee of Universidad Privada Norbert Wiener, under registration number 0017-2025-CIEIC-UPNW, thereby confirming that the research procedures met the required ethical and institutional standards for studies involving human participants.

## 3. Results

### 3.1. Evidence Based on Content

The expert judges evaluated the content of the items according to the criteria of relevance, representativeness, and clarity, with Aiken’s V values exceeding 0.70 (Table 2). These results indicate adequate content validity and acceptable agreement among judges regarding the adequacy of the items. All expert judges (n = 5) approved the final version of the LWMS, including its semantic and contextual suitability for the target population. Likewise, in the pilot sample (n = 10), no suggestions or modifications were made. Feedback obtained through cognitive probing indicated that the items were clearly understood and interpreted as intended, supporting their linguistic naturalness and comprehensibility in the target context.

### 3.2. Descriptive Analysis

Table 3 shows that the highest arithmetic mean was observed for item 1 (M = 2.28), while the lowest was found for item 4 (M = 1.37). With regard to variability, the greatest standard deviation occurred in item 1 and 3 (SD = 0.99) and the smallest in item 4 (SD = 0.69). The skewness and kurtosis values fell within the ±1.5 range, with the exception of item 4. Item 4 showed higher skewness (g_1_ = 1.97) and kurtosis (g_2_ = 3.79), suggesting a distribution skewed toward lower values and a possible floor effect. This indicates a low frequency of the behaviour assessed in the sample, and therefore its interpretation should be made with caution. In addition, all corrected item–total correlations were satisfactory, exceeding 0.30. Finally, the polychoric correlation matrix indicated positive relationships, with no evidence of multicollinearity (r > 0.90).

### 3.3. Evidence Based on Internal Structure

**Figure 1:**
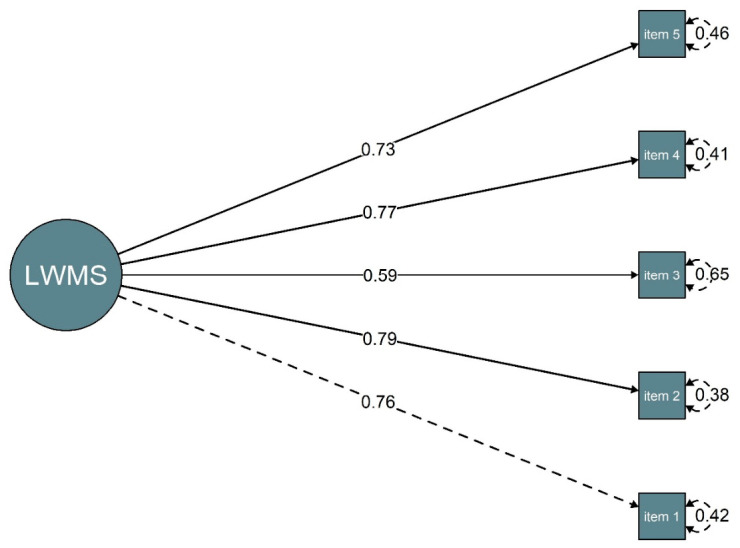
Factor structure of the LWMS.

The confirmatory factor analysis supported a unidimensional structure of the LWMS, showing good model fit (CFI = 0.99, RMSEA = 0.04 [90% CI: 0.001, 0.092], SRMR = 0.02). Standardised factor loadings ranged from 0.59 to 0.79, indicating that all items contributed meaningfully to the latent construct (Figure 1).

### 3.4. Reliability

The posterior estimate of the ω coefficient was 0.79, with a 95% credibility interval ranging from 0.75 to 0.82, indicating a 95% probability that the true value of ω lies within this range. Likewise, the reliability coefficients H = 0.86 and r_xx_ = 0.78 considered adequate.

### 3.5. Evidence Based on the Relationship with Other Variables

**Figure 2:**
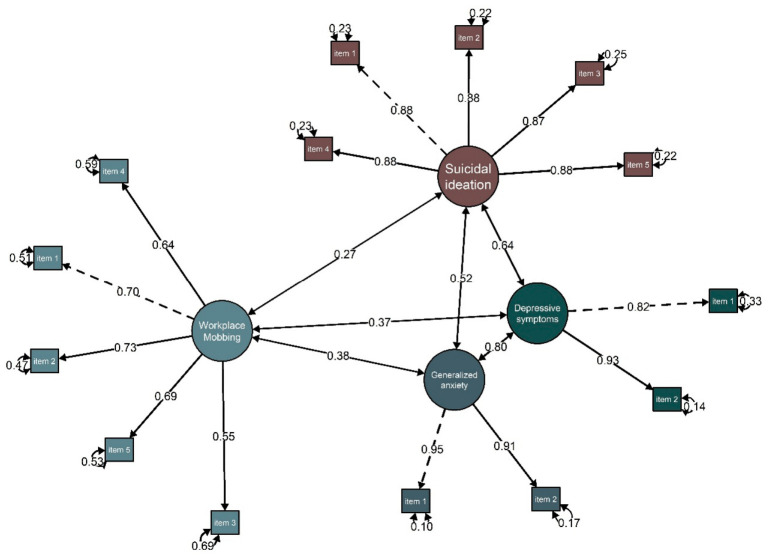
Structural model of the relationship between workplace mobbing, generalised anxiety, depressive symptoms and suicidal ideation.

Figure 2 illustrates the results of the correlation analysis examining the associations between workplace mobbing and the psychological variables included in the model. The estimated structural equation model showed an adequate fit to the data, as indicated by the goodness-of-fit indices (CFI = 0.99, RMSEA = 0.03, [90% CI: 0.005, 0.045], and SRMR = 0.03), supporting the robustness of the proposed relational structure.

The findings revealed that workplace mobbing was positively and statistically significantly associated with all the psychological outcomes examined. In particular, moderate associations were observed with generalised anxiety (r = 0.38, *p* = 0.001) and depressive symptoms (r = 0.37, *p* = 0.001), while a small association was identified with suicidal ideation (r = 0.27, *p* = 0.001).

### 3.6. Item Response Theory

**Figure 3:**
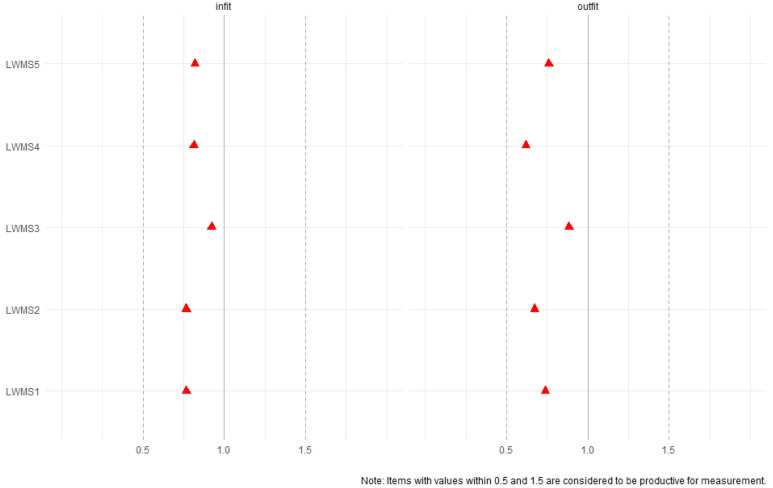
Item Infit and Outfit Statistics of the LWMS.

The assumptions of the model were evaluated and examined. Unidimensionality was supported by the CFA, while local independence was confirmed using the LD-X^2^ index, with standardised associations below 10 (range = −0.148 to 0.132). Regarding monotonicity, no significant violations were identified (crit < 0.40). In addition, infit and outfit values were within ±1.5 (Figure 3).

At the global level, the model demonstrated adequate fit indices (C2 = 6.31, df = 5, RMSEA2 = 0.03 [90% CI: 0.001, 0.083], CFI = 0.99, SRMR = 0.03). To further support these findings, item-level fit was examined in Table 4, where the values indicated excellent fit (RMSEA.S-_X_^2^ < 0.06). With respect to the discrimination parameter (a), item 2 showed the greatest capacity to differentiate levels of the latent trait. Regarding the difficulty parameter (*β*), a progressive increase was observed in the probability of selecting higher response categories as the level of θ increased, as detailed in Table 4.

**Figure 4:**
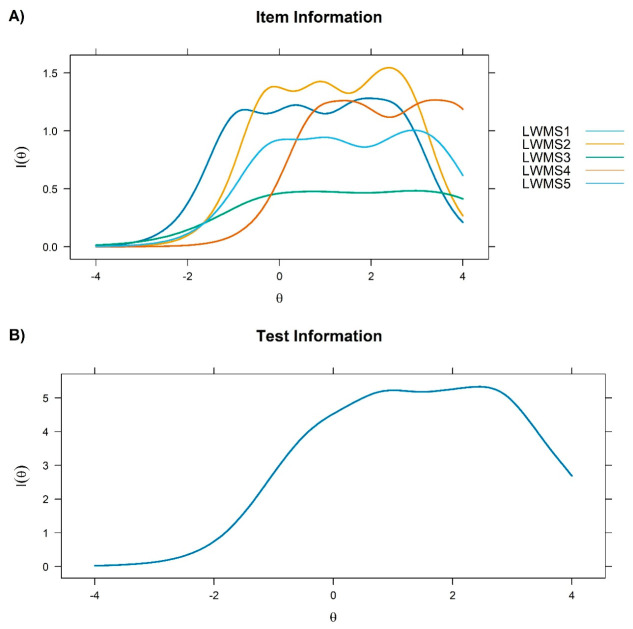
Item information function (**A**) and test information function (**B**) of the LWMS.

Moreover, Figure 4 shows that item 2 provided the greatest amount of information, further highlighting that the scale is particularly precise in assessing high levels of workplace mobbing.

## 4. Discussion

Over recent decades, mobbing has ceased to be conceptualised exclusively as an organisational problem and has instead been recognised as a psychosocial stressor with clinically significant implications for adult mental health (Meng, 2025). Its cumulative, frequently subtle and relational nature requires brief yet psychometrically robust assessment instruments capable of accurately identifying exposure to such behaviours. In this context, the validation of the Spanish version of the LWMS is particularly relevant, as it provides empirical evidence regarding its internal structure, consistency and item-level functioning, thereby strengthening its utility in Spanish-speaking contexts.

The findings of the present study supported a unidimensional factorial structure of the LWMS, consistent with the original validation proposed by Steffgen et al. (2019). This empirical convergence suggests that mobbing may be conceptualised as a global construct reflecting the cumulative experience of hostile behaviours in the workplace. The replication of this structure in an adult Spanish-speaking sample reinforces the cross-cultural stability of the underlying theoretical model and confirms that the five items adequately capture a single latent factor (Steffgen et al., 2019). From a practical perspective, unidimensionality enhances interpretative parsimony and facilitates its application in population-based studies and organisational assessments, allowing comprehensive measurement without fragmenting the phenomenon into subcomponents that could dilute its systemic character.

In terms of reliability, the scale demonstrated adequate coefficients across multiple approaches, including Bayesian omega, coefficient H and the r_xx_ index derived from item response theory. These findings are consistent with the levels of internal consistency reported in the original LWMS study and further reinforce the robustness of the measure (Steffgen et al., 2019). The use of Bayesian estimates constitutes a methodological strength, as it provides more stable parameters that are less dependent on sample size compared with traditional estimations (Pfadt et al., 2022). Likewise, coefficient H indicated satisfactory replicability of the latent construct, suggesting that the mobbing factor is well defined by its indicators. From an item response theory perspective, the r_xx_ index confirmed that the scale possesses sufficient precision to estimate individual differences in exposure to workplace bullying, strengthening confidence in the interpretation of its scores.

The associations observed between mobbing and variables such as generalised anxiety, depressive symptoms and suicidal ideation are consistent with the international literature conceptualising workplace bullying as a chronic interpersonal stressor with a direct impact on psychological well-being (Espinoza Escobar et al., 2026; Rincon-Hoyos et al., 2025). Repeated exposure to experiences of humiliation, exclusion or devaluation may activate sustained stress mechanisms, erode self-esteem and weaken perceptions of personal control (Meng, 2025; Rincon-Hoyos et al., 2025). These processes favour the emergence of anxious and depressive symptomatology, as well as dysfunctional cognitions such as hopelessness and feelings of worthlessness, recognised risk factors for suicidal ideation (Ayala-Burboa et al., 2024; Machado et al., 2021; Pompili et al., 2008). Moreover, deterioration in workplace relationships and perceived social isolation may amplify emotional vulnerability, particularly in contexts where work constitutes a central source of identity and social recognition (Meng, 2025; Tatar & Yüksel, 2019). In this sense, the findings reinforce the relevance of mobbing as a psychosocial determinant of adult mental health.

The item response theory analysis indicated that the LWMS demonstrates greater precision at higher levels of the latent trait, suggesting that the scale is particularly effective in discriminating between individuals with high exposure to workplace bullying. This characteristic is clinically relevant, as it allows more accurate identification of individuals at elevated risk (Steffgen et al., 2019). However, this pattern also implies that the scale shows comparatively lower precision at low or moderate levels of the construct, which should be considered when interpreting scores in general population or early-stage contexts. In practical terms, the LWMS may be especially useful for identifying individuals with pronounced or chronic exposure to mobbing but less sensitive for detecting subtle, emerging, or less frequent forms of workplace bullying. Although item 4 (“How often are you being ridiculed by your colleagues or your superior in front of others?”) presented skewed distribution and kurtosis indicative of a floor effect, its retention is supported by multiple psychometric indicators. The item showed adequate factor loading, satisfactory item–total correlation, strong discrimination in the IRT model, and appropriate fit indices. This suggests that the item is particularly informative at higher levels of the latent trait, capturing more severe manifestations of workplace mobbing. Therefore, its inclusion contributes to the content validity and sensitivity of the scale. In particular, Item 2 (“How often are you ignored by your colleagues or your superior?”) showed the highest discrimination and information parameters. Social exclusion may constitute one of the most central and psychologically harmful cores of mobbing, as it directly threatens the basic need for belonging and interpersonal recognition (Meng, 2025). Unlike overtly aggressive behaviours, being ignored may be more subtle yet profoundly destabilising, generating feelings of invisibility and rejection that affect professional identity (Tatar & Yüksel, 2019). The high discriminative power of this item suggests that the experience of being ignored may function as an early or particularly sensitive indicator of workplace harassment dynamics (Steffgen et al., 2019). From an applied perspective, these findings suggest that the LWMS is particularly suitable for use in contexts where the objective is to identify high-risk cases or more severe manifestations of mobbing (e.g., clinical assessment or organisational risk detection). At the same time, caution is warranted when using the scale as a broad screening tool in low-exposure populations, as its reduced sensitivity at lower trait levels may limit its ability to capture milder or incipient forms of workplace conflict. From both clinical and organisational perspectives, this finding underscores its usefulness as a relevant marker in screening processes and assessments of organisational climate.

The study has both theoretical and practical implications. Theoretically, the findings support the conceptualisation of mobbing as a coherent and unidimensional construct while providing evidence derived from both structural and probabilistic measurement models. The integration of structural equation modelling and item response theory strengthens the instrument’s validity by demonstrating both factorial coherence and item-level precision (Steffgen et al., 2019). From a practical standpoint, the availability of a brief and validated Spanish version of the LWMS facilitates its implementation in organisational, occupational and clinical contexts where assessment time and resources are often limited. Its brevity, combined with evidence of precision and adequate item discrimination, supports its use in both screening processes and broader evaluations of organisational climate and psychosocial risks.

In the Peruvian context, characterised by high levels of labour informality, power asymmetries in employment relationships, and limitations in regulatory and supervisory mechanisms, the availability of a brief and psychometrically sound instrument is particularly relevant (Instituto Peruano de Economía, 2025). However, these implications should be interpreted with caution, as the findings are based on a non-probabilistic sample with relatively high educational attainment and access to digital resources, which may not fully represent more vulnerable or informal sectors of the workforce. The LWMS may contribute to the early identification of workplace bullying situations, facilitating the implementation of evidence-based preventive interventions, the design of organisational policies aimed at promoting healthy work environments, and the development of psychosocial risk monitoring programmes. Furthermore, its systematic use could support decision-making processes in human resources, workplace well-being programmes and expert assessments in legal or administrative contexts. Therefore, its applicability may be particularly relevant for formal employment settings or populations with similar sociodemographic characteristics to those included in the present study. Taken together, these implications position the Spanish version of the LWMS as a relevant tool for research and professional practice in the field of occupational health in Peru, while highlighting the need for further validation in more diverse and underrepresented segments of the workforce.

From a practical perspective, the LWMS represents a brief and efficient tool for the early detection of workplace mobbing in organisational and clinical settings. Its use may facilitate the identification of at-risk individuals, inform preventive interventions, and support decision-making in occupational health policies. Additionally, its brevity makes it particularly suitable for large-scale screenings and applied research contexts. Building on these implications, several practical recommendations can be derived. First, organisations should incorporate brief screening tools such as the LWMS into regular psychosocial risk assessments to enable early detection of mobbing behaviours. Second, intervention programmes should prioritise the reduction in social exclusion dynamics, given their central role in the phenomenon, as identified in the present study. Third, organisations are encouraged to develop clear reporting and response protocols to address workplace bullying, ensuring psychological safety and confidentiality for employees. Finally, policymakers and occupational health practitioners should promote the integration of validated instruments into national guidelines for workplace risk assessment, particularly in contexts characterised by labour vulnerability. These recommendations highlight that the utility of the LWMS extends beyond measurement, serving as a tool to inform prevention strategies, organisational decision-making and the design of healthier work environments.

Among the study’s strengths are the application of Bayesian reliability estimates and the use of item response theory, which enabled a comprehensive evaluation of the instrument beyond traditional analyses and provided new psychometric contributions regarding the LWMS (Steffgen et al., 2019). Nevertheless, the findings should be interpreted in light of certain limitations. First, the use of non-probabilistic sampling restricts the generalisability of the results to the wider population. Moreover, the online recruitment strategy (via social media) implies an unknown response rate and increases the likelihood of self-selection bias, which may have led to the overrepresentation of individuals with a particular interest in workplace experiences or mental health topics. Additionally, although common method bias was assessed using Harman’s single-factor test and did not appear to substantially affect the results, the exclusive use of self-report measures may still introduce shared variance due to factors such as social desirability or measurement context. Second, the cross-sectional design precludes establishing causal relationships between exposure to mobbing and the mental health variables assessed and may further contribute to shared method variance. Additionally, the overrepresentation of participants with university and postgraduate education may introduce biases associated with educational level, affecting sample representativeness and potentially influencing response patterns. Likewise, the sample showed limited occupational diversity, which may restrict the applicability of the findings across different work contexts.

It is important to note that the absence of measurement invariance analyses constitutes a relevant limitation for a validation study. In particular, invariance across gender and other key sociodemographic variables (e.g., educational level or type of employment) was not evaluated, as the sample size is relatively small and the groups are not equivalent, which limits the extent to which the scale can be assumed to function equivalently across groups. Furthermore, within the framework of item response theory, differential item functioning (DIF) across gender was not examined, which would have provided more precise evidence regarding potential item-level bias.

Accordingly, future research should employ longitudinal designs to examine the directionality and stability of the observed associations. It would also be advisable to replicate the study using probabilistic sampling or more heterogeneous sampling strategies to enhance population representativeness. Future studies should also explicitly test measurement invariance (e.g., configural, metric, and scalar invariance) across gender, educational level, and employment conditions to ensure cross-group comparability. Additionally, conducting DIF analyses within an IRT framework would allow for the identification of potential item-level biases and further strengthen the robustness of the instrument. Furthermore, assessing the temporal stability of the instrument through test–retest studies, as well as analysing measurement invariance across different demographic groups, would strengthen the accumulated evidence regarding its validity and reliability. Finally, the inclusion of clinical or specific occupational samples could broaden the instrument’s applied scope and provide further evidence of external validity.

## 5. Conclusions

The Spanish version of the LWMS demonstrates generally adequate psychometric properties, including a consistent unidimensional structure, acceptable reliability, and expected associations with relevant mental health indicators. The scale shows particular precision at higher levels of workplace bullying, highlighting the relevance of the item related to social exclusion as a central indicator of the phenomenon. However, these findings should be interpreted with caution given the use of non-probability sampling and the absence of measurement invariance, test–retest reliability, and differential item functioning analyses. These findings provide preliminary support for its utility as a brief and potentially useful instrument for assessing mobbing in adults. From an applied perspective, the LWMS may serve as a practical tool for early identification of high-risk cases in organisational settings, supporting psychosocial risk assessment and workplace climate evaluations. In clinical contexts, it may assist in the detection of individuals exposed to severe forms of workplace bullying, informing assessment and intervention processes. Additionally, at a policy level, the availability of a validated brief instrument may contribute to the development of evidence-based guidelines and monitoring strategies for psychosocial risks in the workplace.

Overall, the LWMS represents a promising instrument for research and applied use in Spanish-speaking contexts, particularly in settings where brief, reliable, and targeted assessment tools are required, while further studies are needed to strengthen its generalisability and cross-group validity.

## Figures and Tables

**Table 1:** Final Spanish version of the LWMS.

Items	Original Version in English	Spanish Version
1	How often is your work being criticized by your colleagues or your superior?	¿Con qué frecuencia sus colegas o superiores critican su trabajo?
2	How often are you being ignored by your colleagues or your superior?	¿Con qué frecuencia sus colegas o su superior le ignoran?
3	How often are you being assigned absurd duties by your superior?	¿Con qué frecuencia su superior le asigna tareas absurdas?
4	How often are you being ridiculed by your colleagues or your superior in front of others?	¿Con qué frecuencia sus colegas o superiores se burlan de usted delante de otras personas?
5	How often do you have conflicts with your colleagues or your superior?	¿Con qué frecuencia tiene conflictos con sus colegas o con su superior?

**Table 2:** Content validity of the LWMS items.

Items	Relevance (n = 5)		Representativeness (n = 5)		Clarity (n = 5)	
	V	CI 95%	V	CI 95%	V	CI 95%
1	0.93	0.75, 0.99	0.73	0.52, 0.87	0.73	0.52, 0.87
2	0.87	0.67, 0.95	0.93	0.75, 0.99	0.87	0.67, 0.95
3	0.73	0.52, 0.87	0.87	0.67, 0.95	0.93	0.75, 0.99
4	0.93	0.75, 0.99	0.73	0.52, 0.87	0.73	0.52, 0.87
5	0.87	0.67, 0.95	0.93	0.75, 0.99	0.87	0.67, 0.95

**Table 3:** Descriptive measures and correlation matrix.

Items	M	SD	g_1_	g_2_	*r*_it_	Polychoric Correlation Matrix				
1	2.28	0.99	0.52	−0.11	0.61	-				
2	1.88	0.93	0.97	0.70	0.62	0.62	-			
3	1.90	0.99	0.95	0.39	0.49	0.44	0.47	-		
4	1.37	0.69	1.97	3.79	0.55	0.52	0.61	0.46	-	
5	1.80	0.88	0.98	0.76	0.60	0.57	0.53	0.44	0.61	-

**Note**. M = media, SD = standard deviation, g_1_ = skewness, g_2_ = kurtosis, *r*_it_ = corrected item test correlation.

**Table 4:** Discrimination, difficulty, and item fit parameters.

Items	Item Parameters					Item Fit	
	*a*	*β*_1_	*β*_2_	*β*_3_	*β*_4_	*S*-X^2^ (*gl*)	RMSEA.S-_X_^2^
1	2.10	−0.922	0.362	1.659	2.599	18.133 (14)	0.029
2	2.27	−0.271	0.915	2.145	2.747	16.669 (13)	0.029
3	1.26	−0.269	1.060	2.708	3.723	10.166 (17)	0.001
4	2.08	0.788	1.734	3.072	3.992	14.608 (12)	0.025
5	1.83	−0.186	1.104	2.637	3.359	5.635 (12)	0.001

**Note**. a = discrimination parameter. *β* = difficulty parameter. S-X^2^ = fit index. df = degrees of freedom. RMSEA.S-_X_^2^ = root mean square error of approximation.

## Data Availability

The data presented in this study are available upon request from the corresponding author due to privacy issues.
